# Long-term survival of HER2 positive gastric cancer patient with multiple liver metastases who obtained pathological complete response after systemic chemotherapy: A case report

**DOI:** 10.1016/j.ijscr.2022.107097

**Published:** 2022-04-20

**Authors:** Masaki Sakaue, Keijiro Sugimura, Toru Masuzawa, Atsushi Takeno, Shinnsuke Katsuyama, Go Shinnke, Ryo Ikeshima, Kenji Kawai, Masayuki Hiraki, Yoshiteru Katsura, Yoshiaki Ohmura, Taishi Hata, Yutaka Takeda, Kohei Murata

**Affiliations:** Department of Surgery, Kansai Rosai Hospital, 3-1-69, Inabaso, Amagasaki city, Hyogo 660-8511, Japan

**Keywords:** Gastric cancer, Liver metastasis, Conversion surgery, Pathological complete response

## Abstract

**Introduction and importance:**

Advanced gastric cancer with liver metastasis is classified as stage IV disease and is generally treated with systemic chemotherapy. Despite recent advances in chemotherapy regimens, the prognosis for gastric cancer with liver metastasis is poor. Recent studies reported the effectiveness of upfront chemotherapy followed by conversion surgery for gastric cancer with liver metastasis. Here, we report a case of an advanced stage IV gastric cancer with liver metastasis treated with upfront systemic chemotherapy followed by conversion surgery, which resulted in pathological complete response and good prognosis.

**Case presentation:**

A 79-year-old man diagnosed with human epidermal growth factor receptor type 2 (HER2)-positive gastric cancer with multiple liver metastases. He underwent systemic chemotherapy with capecitabine, cisplatin, and trastuzumab. After 14 courses of chemotherapy, the primary tumor and liver metastases shrank, suggesting a partial response. We performed distal gastrectomy with D2 dissection plus lateral hepatic segment resection. Pathological examination revealed no residual tumor cells in the primary or metastatic sites, which indicated a pathological complete response. The postoperative course was uneventful. The patient was discharged on postoperative day 8. Adjuvant S-1 chemotherapy was started on postoperative day 46 and given for 1 year. The patient has been alive and recurrence-free for approximately 5 years after surgery.

**Conclusion:**

This case shows the possibility of conversion surgery after systemic chemotherapy for stage IV advanced gastric cancer with liver metastasis.

## Highlights

• Conversion surgery was underwent for HER2 positive gastric cancer patient with multiple liver metastases.
• Pathological complete response and long-term survival without recurrence was obtained.
• Systemic chemotherapy consisting of capecitabine, cisplatin and trastuzumab was performed.

## 1 Introduction

Gastric cancer is the fifth most common carcinoma and the third leading cause of death in the world [1]. Simultaneous liver metastasis occurs in 3% to 14% of all gastric cancers [2], [3]. Simultaneous liver metastasis of gastric cancer is considered to be distant metastasis. It is considered a contraindication for surgical resection and an indication for systemic chemotherapy. Despite advances in chemotherapy regimens, the prognosis for gastric cancer with liver metastasis is poor. In general, with chemotherapy alone, median survival is considered to be 10 to 15 months and the 3-year survival rate is approximately 10% [4], [5], [6].

Another treatment option for stage IV gastric cancer is systemic chemotherapy followed by conversion surgery. With the recent progress in the development of anti-cancer drug treatments including molecularly targeted drugs, the number of cases in which even stage IV gastric cancer can be resected without any residue is increasing due to better therapeutic effect. Furthermore, gastric cancer with liver metastasis has a good prognosis when conversion surgery was performed [7], [8], [9], [10], [11], [12].

Here, we report a case of gastric cancer with multiple liver metastases treated with upfront systemic chemotherapy followed by conversion surgery. The patient had pathological complete responses for the primary and metastatic lesions, resulting in long-term survival. This case report has been reported in line with the SCARE 2020 criteria [13].

## 2 Case presentation

**Fig. 1:**
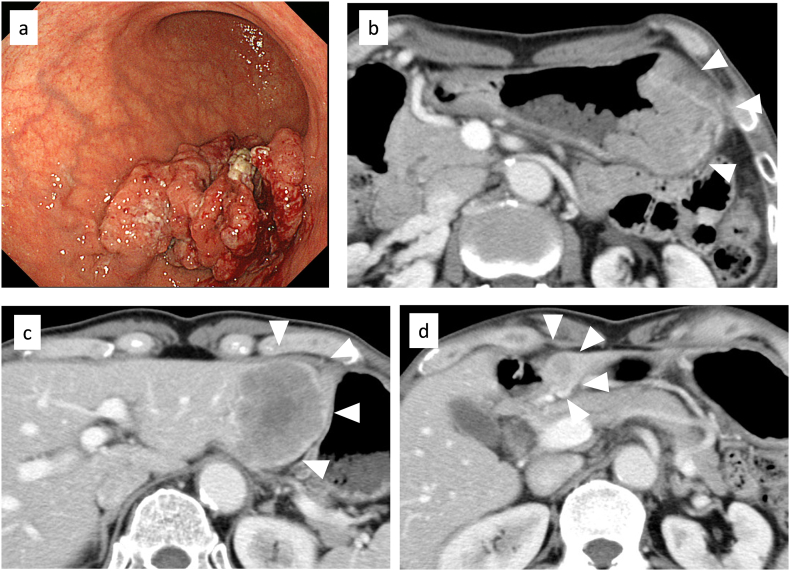
Esophagogastroduodenoscopy (EGD) and computed tomography (CT) findings before chemotherapy. (a) EGD revealed Borrmann type 3 cancer in the lower part of the gastric corpus between the greater curvature and the anterior wall. Abdominal CT showed (b) a thickened gastric wall and multiple liver metastases in (c) S2/3 and (d) S3.

**Fig. 2:**
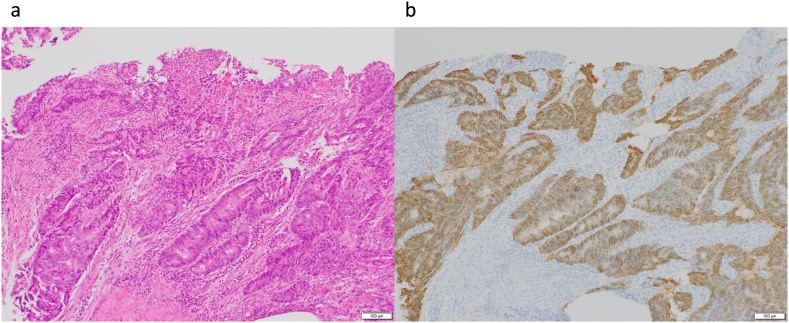
(a) Hematoxylin and eosin staining of the biopsy specimen confirmed well differentiated adenocarcinoma. (b) Human epidermal growth factor receptor type 2 (HER2) immunohistochemical staining in the biopsy specimen confirming strong expression of HER2 protein in tumor cells.

A-79-year-man who had been followed for diabetes mellitus by our hospital's internal medicine department was referred to our gastroenterological surgery department for progressive anemia. Physical examination showed no palpable masses in the abdomen or superficial lymphadenopathy. Blood tests revealed a low hemoglobin level (10.1 g/dl). Other serum biochemical tests indicated normal hepatobiliary function, renal function, carcinoembryonic antigen levels, and serum carbohydrate 19–9 levels. Esophagogastroduodenoscopy showed a Borrmann type 3 tumor in the lower part of the gastric corpus between the greater curvature and the anterior wall (Fig. 1a). Abdominal contrast-enhanced computed tomography revealed a thickened gastric wall and two swollen lymph nodes along with the greater curvature. Multiple liver metastases were also detected (50 mm in S2/3, 19 mm in S3, and 7 mm in S8) (Fig. 1b–c). Ascites was not observed. Biopsy confirmed well differentiated adenocarcinoma and a cluster of tumor cells with strong basolateral membranous reactivity to human epidermal growth factor receptor type 2 (HER2) in immunohistochemical staining (Fig. 2a–b). We diagnosed unresectable gastric cancer (Borrmann type 3, cT3, cN2, cH1, cP0, cM1(H), cStage IV), according to the eighth edition of the UICC guidelines.

**Fig. 3:**
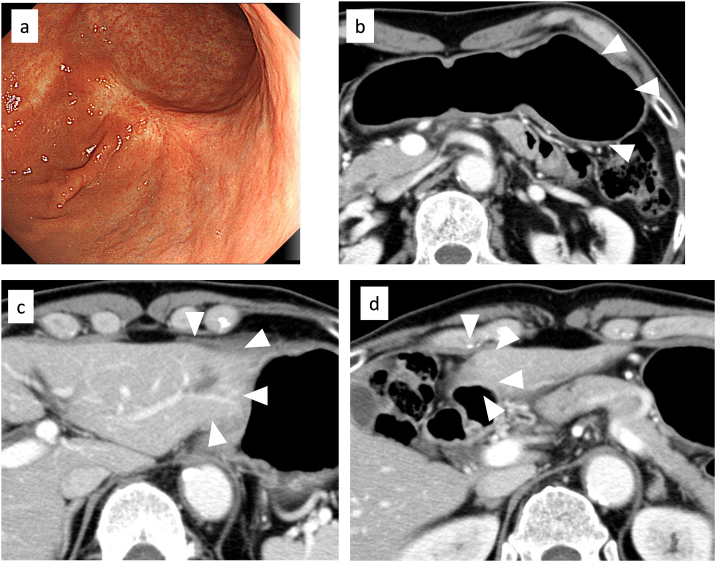
Esophagogastroduodenoscopy (EGD) and computed tomography (CT) findings after chemotherapy. (a) EGD revealed shrinkage of primary tumor. (b–d) Abdominal CT showed shrinkage of the primary tumor and metastatic liver tumors.

**Fig. 4:**
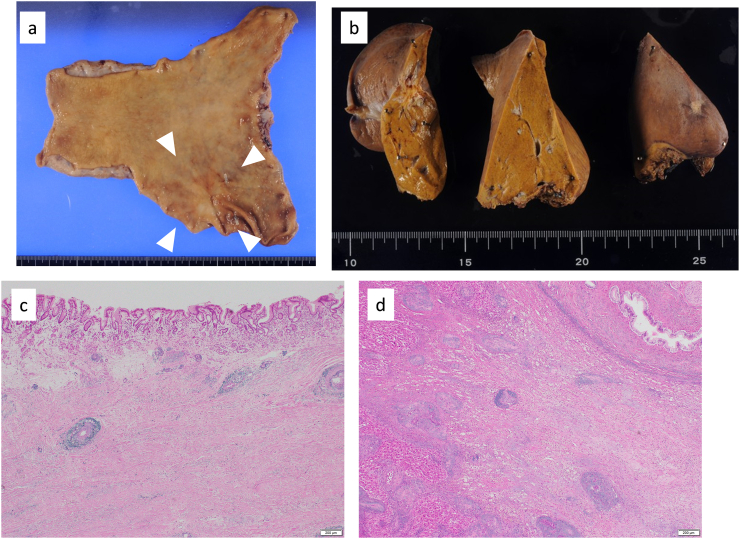
Surgically resected specimen of the primary tumor and liver metastases. (a) Macroscopic examination showed only residual scar tissue and no obvious neoplastic lesions in the primary gastric lesion. (b) Macroscopic examination showed only residual scar tissue in the left lateral segment of the liver. Pathological examination revealed no residual tumor cells in (c) the resected primary gastric lesion and (d) the resected left lateral segment of the liver.

Chemotherapy with capecitabine (1500 mg/body/day on days 1–14), cisplatin (80 mg/m^2^ on day 1), and trastuzumab (6 mg/kg on day 1) was initiated. The chemotherapy regimen was repeated every 3 weeks. Follow-up esophagogastroduodenoscopy after 14 courses demonstrated that the main gastric lesion and metastatic lymph nodes were less than half as large (Fig. 3a–b). The metastatic liver lesions also shrank to less than half of their size before chemotherapy (Fig. 3c–d). We thought that R0 resection was possible and considered conversion surgery. Laparoscopic examination showed no peritoneal metastases. Peritoneal lavage cytology showed no cancer cells in the abdominal cavity. We performed distal gastrectomy with D2 lymph node dissection and Billroth I reconstruction plus left lateral hepatic segmentectomy. The S8 tumor identified before treatment could not be detected during the operation, so the site was not resected. Operative time was 305 min and intraoperative blood loss was 153 ml. Macroscopic examination showed only residual scar tissue and no obvious neoplastic lesions in the primary gastric lesion (Fig. 4a–b). Pathological examination revealed no residual tumor cells in the resected primary gastric lesion (Fig. 4c). No tumor cells were found in the lymph nodes or the resected liver specimen (Fig. 4d). The therapeutic effect of chemotherapy was grade 3, which corresponded to a complete response according to the 14th Japanese guidelines on gastric cancer.

The postoperative course was uneventful. The patient was discharged on postoperative day 8. Adjuvant oral S-1 chemotherapy was given for 1 year after surgery. The patient has remained disease-free for 5 years after surgery.

## 3 Discussion

Gastric cancer with distant metastases such as peritoneal metastasis, liver metastasis, and paraaortic lymph node metastasis is considered to be stage IV gastric cancer. The prognosis is extremely poor. However, with the recent development of chemotherapy including molecularly targeted drugs, some reports have suggested that prognosis can be improved if disease control is obtained by chemotherapy and R0 resection can be performed with conversion surgery. However, there are many unclear points, such as the indications and timing for conversion surgery; it is currently controversial. Phase III trials to verify the effectiveness of conversion surgery for stage IV gastric cancer are currently in progress and we should wait for the results.

Oki et al. reported the results of surgical treatment after chemotherapy for patients with gastric cancer and liver metastasis. [10] They investigated 69 patients who underwent surgical treatment for liver metastasis after chemotherapy. The median survival was 3.4 years (40.8 months) and recurrence-free survival was 11.3 months, which suggested that these patients have better survival than patients with stage IV gastric cancer in general, who have median survival of 9.5–14.1 months. In addition, among patients that underwent resection, a single liver metastasis and few lymph node metastases were cited as good prognostic factors. Such patients are considered to be good candidates for conversion surgery. Takemura et al. examined 64 patients who underwent resection for gastric cancer with simultaneous or metachronous liver metastasis [14]. They found that serosal invasion by the primary tumor and diameter of the largest liver metastasis > 5 cm are poor prognostic factors. Thus, patients without those factors are good candidates for conversion surgery. Until now, only one prospective study by Fujitani et al. has investigated the usefulness of conversion surgery for gastric cancer with liver metastasis. [15] In their study, 70 patients with simultaneous or metachronous liver metastasis from gastric cancer were treated with upfront chemotherapy. The 48 patients who had a therapeutic effect underwent conversion surgery, of whom 43 achieved R0 resection. Although their study reported only short-term results, the complication rate was 27.9% and short-term results were sufficiently acceptable. In the future, information on long-term prognosis is awaited.

The key cytotoxic drugs used as first-line chemotherapy for advanced gastric cancer are pyrimidine fluoride, platinum, taxanes, and irinotecan. The molecular target agent for HER2 positive gastric cancer is the anti-HER2 antibody trastuzumab. Since this patient had HER2-positive gastric cancer, treatment was started with capecitabine, cisplatin, and trastuzumab according to a previously described treatment strategy [16]. In patients with stage IV gastric cancer, there are many unclear points regarding optimal treatment duration from the start of chemotherapy to conversion surgery. Chen et al. analyzed 95 patients with stage IV gastric cancer who underwent conversion surgery after chemotherapy to evaluate the optimal treatment duration [17]. Their results indicated that patients who received 6 or more cycles had better prognosis than patients who received 5 or fewer cycles. Thus, they concluded that 6 or more cycles of induction chemotherapy are recommended. In our patient, 14 courses were given; the treatment duration might have been sufficient.

A recent analysis of patients who underwent upfront chemotherapy followed by conversion surgery for gastric cancer with liver metastasis identified pathological T stage, pathological N stage, size of liver metastases, number of liver metastases, presence of both lobes, R0 resection, clinical response, and presence of other non-curable factors as prognostic factors [7], [8], [9], [10], [14], [15]. On the other hand, the therapeutic effect on the pathological primary lesion or metastatic lesions and the effect on prognosis have not been fully investigated. Kinoshita et al. examined 57 cases of stage IV gastric cancer including liver metastasis in patients who underwent upfront triplet chemotherapy followed by conversion surgery [8]. They found that pathological complete response of the primary lesion occurred in 2 cases (5.9%), which is a low frequency. In our case, pathological compete response was obtained in the primary lesion, lymph nodes, and liver metastases, which is considered to be extremely rare. Furthermore, it is noteworthy that recurrence-free survival has been sustained for a long period, more than 5 years. In the future, it might be possible to examine whether pathological therapeutic effect is associated with prognosis in a comprehensive case study.

## 4 Conclusions

In conclusion, we report a case of successful systemic chemotherapy with trastuzumab followed by conversion surgery for stage IV gastric cancer with multiple liver metastases. Pathological examination showed no residual tumor cells in the gastric and metastatic lesions. Further evidence and prospective clinical trials are essential to establish the optimal strategy for stage IV gastric cancer with liver metastasis.

## Abbreviation

HER2: human epidermal growth factor receptor type 2

## Provenance and peer review

Not commissioned, externally peer-reviewed.

## Consent

The patient in this case study provided written informed consent. Written informed consent was obtained from the patient for publication of this case and any accompanying images.

## Ethical approval

The study was approved by the human ethics review committee of Kansai Rosai Hospital and conducted in accordance with the Declaration of Helsinki.

## Funding

The authors declare that they received no funding for this work.

## Guarantor

Keijiro Sugimura.

## Research registration number

None.

## CRediT authorship contribution statement

Sakaue and Sugimura designed and directed the project and wrote the manuscript with support from the other authors. All authors discussed the results and contributed to the final manuscript.

## Declaration of competing interest

All authors have no conflicts of interest to declare. All authors have no disclosures of funding to declare.
